# Development of an Instrumented Climbing Hold with an Embedded Six-Axis Force Sensor for Speed Climbing

**DOI:** 10.3390/s26103220

**Published:** 2026-05-19

**Authors:** Akihiro Kawamura, Takumi Shintani, Shimpei Aihara, Ryo Kurazume

**Affiliations:** 1 Faculty of Information and Electrical Engineering, Kyushu University, Fukuoka 819-0395, Japan; kurazume@ait.kyushu-u.ac.jp; 2 Department of Sport Science, Japan Institute of Sports Sciences, Tokyo 115-0056, Japan; shimpei.aihara@jpnsport.go.jp; 3 Graduate School of Information and Electrical Engineering, Kyushu University, Fukuoka 819-0395, Japan; shintani.takumi.183@s.kyushu-u.ac.jp

**Keywords:** speed climbing, force measurement, instrumented climbing hold, six-axis force sensor, biomechanical analysis, sports engineering

## Abstract

Understanding the interaction forces between climbers and climbing holds is important for motion analysis and performance evaluation in sport climbing. In particular, force measurement during speed climbing can provide valuable insights into explosive movements and athlete performance. However, many existing measurement systems require modifications to the climbing wall structure or sensors installed behind the wall, which limits their applicability to existing speed climbing facilities. This study proposes a wireless instrumented climbing hold for speed climbing that enables force-related measurement without modifying the wall structure. The proposed system integrates a six-axis force sensor, a microcomputer, a wireless communication module, and a battery inside the climbing hold. This self-contained configuration allows the hold to wirelessly transmit force and moment data during climbing while maintaining compatibility with standard speed climbing walls and competition environments. In addition, the system enables an estimation of the point of force application on the hold surface by combining measured force and moment data with the three-dimensional hold geometry. Experimental evaluations were conducted to verify the feasibility and performance of the system. External load tests using a digital force gauge confirmed that the embedded sensor can measure static loads and respond to rapidly changing loads with sufficient temporal responsiveness, and the estimated point of force application corresponded closely to the actual loading point. Furthermore, measurements on an actual speed climbing wall demonstrated that the proposed system can successfully capture interaction forces during climbing movements. These results indicate that the proposed system is a practical tool for force-based motion analysis in speed climbing.

## 1. Introduction

Sport climbing is a competitive discipline in which athletes ascend artificial walls by grasping and stepping on protrusions called holds. It consists of three disciplines: Boulder, Lead, and Speed. In the Boulder discipline, athletes attempt to complete short routes, and their performance is evaluated based on the number of problems completed and the number of attempts required. In the Lead discipline, athletes are ranked according to the highest point they reach on a route. In the Speed discipline, athletes compete to reach the top of the wall in the shortest possible time. In recent years, speed climbing has attracted increasing attention as an Olympic event. At the Paris 2024 Olympic Games, speed climbing was contested as a standalone medal event, separate from the boulder-and-lead combined format.

**Figure 1:**
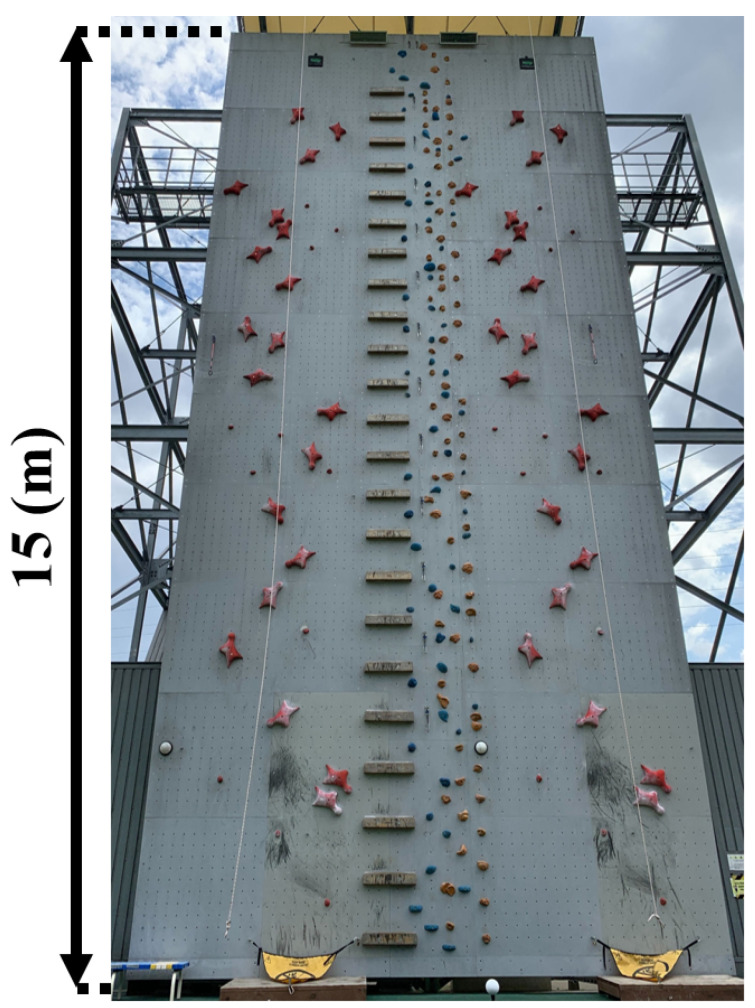
Speed wall.

**Figure 2:**
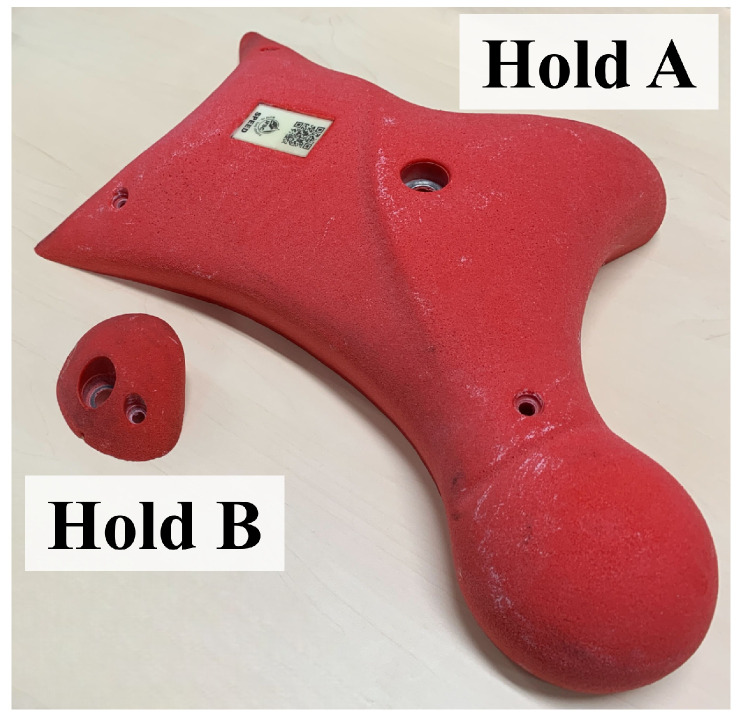
Two types of holds. Hold A is mainly used by the hands, while Hold B is mainly used by the feet. However, no restrictions are imposed on the use of either hold.

Among the three disciplines, speed climbing is performed on a standardized wall in which the positions and orientations of the holds are fixed, and the route is composed of only two types of holds, as shown in Figure 1 and Figure 2. This standardized environment makes it possible to compare movement and force generation under highly similar conditions. At the same time, performance depends strongly on explosive movement and the efficient generation of force during the start and early acceleration phases [1,2,3,4,5].

Although speed-climbing-specific training and coaching methods have been practiced, there have been relatively few examples of quantitative evaluation in speed climbing, and many existing studies have mainly relied on kinematic analysis [1,2,5]. Typical approaches focus on body kinematics, such as the motion of the pelvis or the center of mass, using image analysis or motion capture. By contrast, fewer studies have directly measured the forces applied to the holds, even though these forces can be regarded as one of the primary sources generating the observed motion.

Understanding the interaction forces between climbers and holds is important for analyzing athlete motion and improving training methods. Several force measurement systems for climbing holds have been proposed in sport climbing [6,7,8,9,10]. These studies have shown that force measurement can provide useful information on climbing technique and movement strategy that is difficult to infer from motion data alone. However, many of the existing systems require modification of the wall structure or installation of sensors behind the wall, which makes them difficult to apply to existing speed climbing walls [6,7,8,9].

This limitation is particularly important in speed climbing, where compatibility with existing competition environments is essential. To address this issue, this study proposes an instrumented climbing hold designed for force-related measurement in speed climbing. The proposed system integrates a six-axis force sensor, a microcomputer, a wireless communication module, and a battery inside the hold, enabling the measurement of forces and moments applied during climbing without modifying the wall structure. In addition, the system enables an estimation of the point of force application on the hold surface by combining measured force and moment data with the three-dimensional geometry of the hold.

The aim of this study is to develop and validate a practical instrumented climbing hold system for speed climbing. Specifically, the study focuses on three objectives: (1) to develop a self-contained wireless hold system that can be installed on an existing speed climbing wall without structural modification or external wiring around the hold, (2) to verify the validity of the structural design and force measurement performance under controlled conditions, and (3) to demonstrate the feasibility of measuring interaction forces on an actual speed climbing wall. The novelty of the proposed system is not limited to the use of a six-axis force sensor or to compatibility with existing walls. Rather, it lies in the integration of six-axis force/moment measurement, point-of-force-application estimation, and battery-powered cable-free operation into a self-contained climbing hold that preserves compatibility with standard speed climbing walls. Through these investigations, this study seeks to establish a basis for force-based motion analysis in speed climbing.

## 2. Related Work

Previous studies on sport climbing have shown that climbing performance is influenced by multiple factors, including strength, endurance, coordination, and movement strategy [11,12,13,14]. At the same time, earlier review studies have pointed out the need for discipline-specific and ecologically valid evaluation methods, because the determinants of performance differ depending on the climbing discipline and measurement environment [12,13].

Compared with Boulder and Lead, speed climbing has been less extensively studied. Nevertheless, its standardized wall and fixed route make it particularly suitable for reproducible biomechanical analysis [2,3]. Fuss and Niegl presented a biomechanical discussion of speed climbing dynamics [3]. More recently, Wolf et al. reported initial findings on accelerating forces during the starting phase [1], and Askari Hosseini and Wolf reviewed performance indicators in speed climbing by combining findings from the literature with video analysis and expert interviews [2]. These studies suggest that the start and early acceleration phases are particularly important for performance, but a comprehensive framework for direct force-based evaluation in actual speed-climbing settings is still lacking [1,2,4,5].

To quantitatively evaluate climbing movement, several sensor-based systems have also been proposed in sport climbing. Fuss and Niegl developed instrumented climbing holds using force transducers and showed that force information and center-of-pressure trajectories can provide useful insight into climbing performance [6]. Iguma et al. proposed a synchronized three-dimensional motion and force measurement system [7], and Colombo et al. developed a sensorized climbing wall for quantitative analysis in a more natural climbing environment [8]. Maffiodo et al. also reported a force-sensing climbing hold capable of real-time multiaxial force measurement and data processing [9]. These studies have shown that force measurement can reveal important aspects of climbing movement that are difficult to infer from motion data alone.

More recently, bolt- and screw-type sensing approaches have been proposed as less intrusive methods for measuring forces applied to climbing holds. In our previous work, a bolt-type force sensor with improved wiring was developed for force measurement in sport climbing [15]. Pernus Weber et al. also proposed an instrumented mounting screw based on strain gauges bonded to a modified mounting screw [10]. These approaches are advantageous because they can reduce the need for wall modification by instrumenting the mounting element itself. However, strain-gauge-based bolt or screw sensors generally require external signal conditioning and data acquisition devices, and the installation of multiple sensors on a full-scale climbing wall may require careful routing of sensor wiring and placement of measurement hardware. This can be a practical limitation when extending the system to a 15-m standard speed climbing wall, especially for holds located high on the wall. In addition, these systems are primarily designed to measure force through the mounting element and do not directly measure three-axis moment components or estimate the point of force application on the hold surface.

Despite these advances, most existing measurement systems have been designed for general sport climbing rather than for speed climbing. Speed climbing differs from the other disciplines in that the route is fixed, the wall configuration is standardized, and the movement is highly explosive and time-constrained [2]. Therefore, a dedicated measurement framework that reflects these characteristics is required. The present study addresses this need by developing a self-contained wireless instrumented hold that integrates a six-axis force sensor, microcomputer, wireless communication module, and battery inside the hold. This self-contained wireless configuration is particularly useful for deployment on a full-scale 15-m standard speed climbing wall, where long sensor cables, wall-side measurement hardware, and wiring routes can become practical constraints, especially for holds located high on the wall. This configuration enables force and moment measurement, point-of-force-application estimation, and force data acquisition during actual speed climbing without modifying the wall structure.

Table 1 summarizes the main characteristics of representative climbing force measurement systems. The comparison highlights the distinctive features of the proposed system, particularly its applicability to existing standard speed climbing walls without structural modification, cable-free operation during climbing, force and moment measurement, and point-of-force-application estimation.

## 3. System Overview

### 3.1. Configuration of the Instrumented Hold

**Figure 3:**
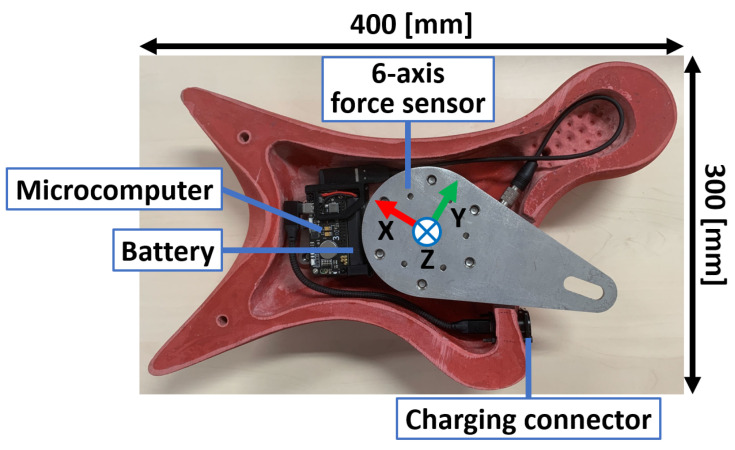
Configuration of the instrumented climbing hold system.

**Figure 4:**
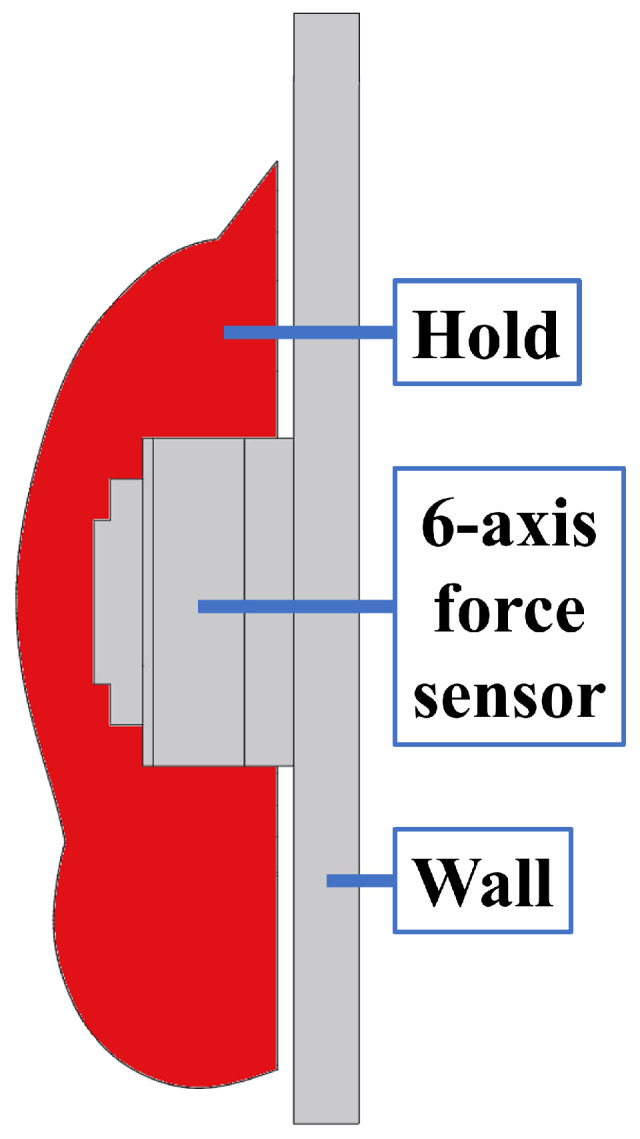
Cross-sectional view of the proposed instrumented climbing hold system.

Figure 3 shows the configuration of the proposed instrumented climbing hold. Figure 4 shows a cross-sectional view of the system. Because a standard climbing hold does not originally contain sufficient internal space for the sensing and electronic components, the interior of the hold was machined to create the required space for embedding a six-axis force sensor, a Raspberry Pi Zero 2 W microcomputer (Raspberry Pi Ltd., Cambridge, UK), a wireless communication module, and a battery. All sensing, computing, and power supply components are integrated inside the hold, allowing the system to be installed on an existing speed climbing wall without modifying the wall structure.

The six-axis force sensor used in this study was manufactured by Leptrino Co., Ltd. (Saku, Japan). Although its metrological characteristics are equivalent to those of an existing six-axis force sensor, its external shape and mechanical housing were customized for integration into the climbing hold. To improve reproducibility and allow comparison with similar commercially available load cells, the manufacturer-specified metrological characteristics of the embedded sensor are summarized in Table 2. These values define the physical measurement range and basic sensing performance of the system used in the present experiments.

The six-axis force sensor is placed between the hold and the supporting fixture so that the external forces and moments applied during climbing are transmitted through the sensor. The force data are sampled at 1000 Hz, and the practical force measurement range of the system is up to 1500 N. The transmitted data are recorded on the external PC with timestamps for synchronization. This self-contained configuration eliminates the need for complex external wiring and improves practicality in actual climbing environments.

To ensure that all loads applied to the hold are measured by the sensor, a clearance is intentionally introduced between the hold and the wall. This prevents direct mechanical contact between the hold and the wall, which would otherwise create load paths that bypass the sensor and reduce measurement accuracy. The required clearance was determined by simulating the expected loading conditions using Autodesk Fusion CAD software (Autodesk Inc., San Francisco, CA, USA, version 2702.1.58) so that sufficient separation could be maintained even under actual climbing loads.

**Figure 5:**
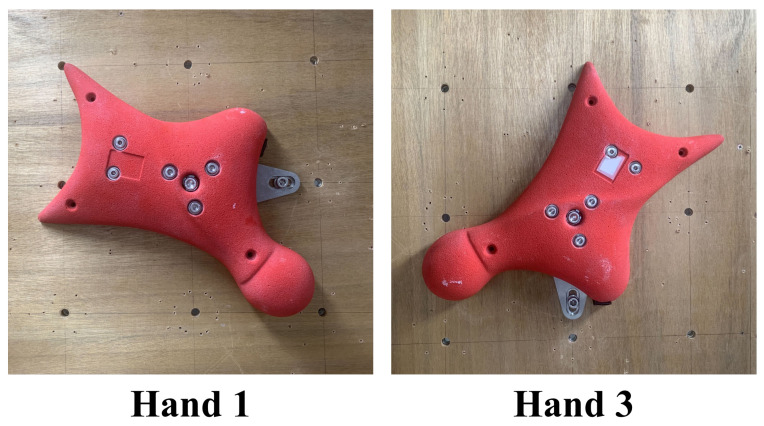
Instrumented hand holds used in this study: Hand 1 and Hand 3.

In a conventional speed climbing wall, a hold is generally fixed using one bolt and three screws. In the proposed system, however, the fixation method was modified to use two bolts in order to improve structural strength and rigidity. Owing to this reinforcement, part of the supporting fixture becomes visible outside the hold. Nevertheless, the fixture was designed so that the exposed portion protrudes only into regions that are not used by speed climbers, thereby minimizing interference with climbing motion and performance. Because the position and inclination of each hold differ depending on its location on the wall, the geometry of the supporting fixture must be adjusted accordingly. In the present study, the first and third hand holds were instrumented. As shown in Figure 5, the locations of the protruding portions differ between these holds, reflecting differences in their mounting conditions and orientations. This hold-specific design enables the system to maintain both mechanical strength and usability while preserving compatibility with the standardized competition wall.

### 3.2. Installation Procedure

**Figure 6:**
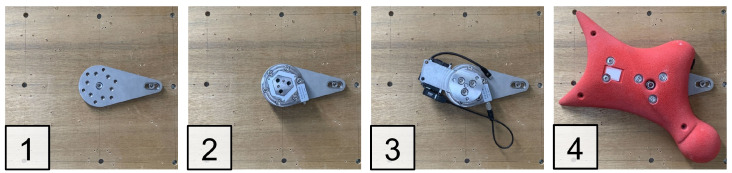
Installation procedure of the proposed instrumented climbing hold system.

Figure 6 shows the installation procedure as follows:

1. Attach the lower attachment to the wall.
2. Install the six-axis force sensor.
3. Attach the upper structure containing electronics.
4. Mount the climbing hold.

This modular design enables installation on an existing speed climbing wall.

### 3.3. Estimation of the Point of Force Application

The proposed system enables estimation and visualization of the point of force application on the hold from the measured force and moment data. Because the embedded six-axis force sensor measures both force and moment components, the point at which the external force is applied can be inferred from these measurements in combination with the three-dimensional geometry of the hold. Let $F={\left[F_{x},F_{y},F_{z}\right]}^{⊤}$ denote the measured force vector, $M={\left[M_{x},M_{y},M_{z}\right]}^{⊤}$ the measured moment vector, and $r={\left[r_{x},r_{y},r_{z}\right]}^{⊤}$ the position vector from the origin of the sensor coordinate system to the point of force application. These quantities satisfy the following relationship:

(1) M=r×F.

Expanding Equation (1) in component form yields

(2) $$\left\{\begin{array}{cc} M_{x} & =r_{y}F_{z}-r_{z}F_{y},\\ M_{y} & =r_{z}F_{x}-r_{x}F_{z},\\ M_{z} & =r_{x}F_{y}-r_{y}F_{x}. \end{array}\right.$$

Equation (2) represents the exact consistency condition between the measured force and moment vectors and the point of force application. In principle, the point of force application can be obtained by solving this set of equations for r.

However, in practice, the measured force and moment data include sensor noise and measurement errors. As a result, Equation (2) may not have an exact solution. To address this issue, a tolerance parameter *t* is introduced, and the condition is relaxed as follows:

(3) $$\left\{\begin{array}{cc} \left|M_{x}-\left(r_{y}F_{z}-r_{z}F_{y}\right)\right| & \leq t,\\ \left|M_{y}-\left(r_{z}F_{x}-r_{x}F_{z}\right)\right| & \leq t,\\ \left|M_{z}-\left(r_{x}F_{y}-r_{y}F_{x}\right)\right| & \leq t. \end{array}\right.$$

Equation (3) defines a feasible set of points that satisfy the force–moment relationship within the tolerance *t*. As *t* increases, the constraints are gradually relaxed, and a non-empty solution region can be obtained even when the measurements are affected by noise or inconsistency.

**Figure 7:**
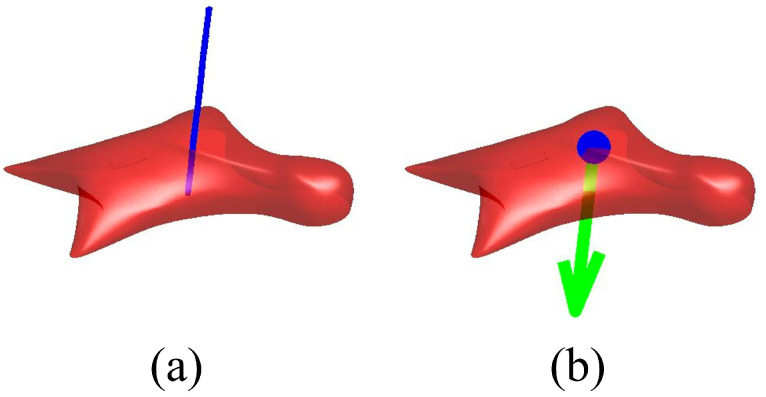
Visualization of the point-of-force-application estimation procedure. (**a**) Possible region of the point of force application. The blue line indicates the rod-like solution region. (**b**) Estimated point of force application on the hold surface. The blue marker indicates the estimated point, and the arrow indicates the measured force vector.

In the present study, the measured values of the six-axis force sensor are substituted into Equation (3), and the unknown position vector r is solved while gradually increasing *t*. This procedure yields a rod-like solution region extending along the line of action of the measured force. The value of *t* is determined by a binary search so that the solution region has a thickness of approximately two to three voxels (about 2–3 mm). This thickness was selected to keep the region as thin as possible while ensuring that its intersection with the hold surface does not vanish. The resulting region is defined as the possible region of the point of force application, as shown in Figure 7.

The estimation procedure is based on the assumption that the point of force application exists on the surface of the hold. Accordingly, the possible region obtained in the above step and the three-dimensional surface model of the hold are voxelized, and their intersection is extracted as the feasible region on the hold surface. The center point of this intersection region is then calculated and defined as the estimated point of force application, as shown in Figure 7.

## 4. Experimental Evaluation

### 4.1. Structural Analysis

An experiment was conducted to verify the validity of the clearance setting between the hold and the wall. In the proposed system, the clearance should be kept as small as possible, because an excessively large clearance may give climbers an unnatural feeling when they touch or pull the hold during climbing. At the same time, a certain clearance is required to prevent direct mechanical contact between the hold and the wall, which would otherwise create load paths bypassing the sensor and reduce measurement accuracy. Therefore, the clearance design must satisfy both requirements: maintaining practical usability for climbers and preserving the validity of force measurement.

**Figure 8:**
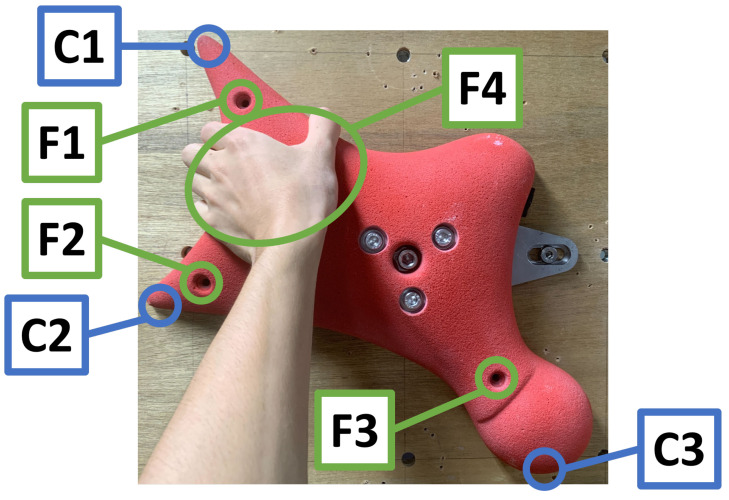
Measurement points for evaluating the clearance between the hold and the wall.

As shown in Figure 8, the clearances were measured at three points, denoted as C1, C2, and C3, while external loads were applied at points F1–F4 to evaluate the resulting deformation. Loads of 50 N were applied at F1–F3 in the direction pushing the hold toward the wall, whereas a load of 150 N was applied at F4 in the direction pulling the hold away from the wall. These loading conditions were selected to evaluate the clearance design under controlled conditions using the available experimental setup.

In actual speed climbing, the hold is primarily subjected to pulling forces toward the climber. Therefore, particular attention should be paid not only to whether direct contact occurs under loading, but also to whether the clearance remains sufficiently small for practical use. The initial clearances without external loading were 1.4 mm at C1, 2.3 mm at C2, and 2.7 mm at C3, and the minimum clearances observed under each loading condition are summarized in Table 3. In Table 3, the loading direction for each condition is indicated, and the values in parentheses represent the change relative to the initial clearance.

Usability was evaluated using three questionnaire items rated on a five-point scale, where higher scores indicated lower perceived interference during use. Questionnaire-based feedback from eight competition-level athletes showed highly favorable usability ratings. The mean ± standard deviation scores were 4.875 ± 0.331 for the perceived difference from a conventional hold, 5.000 ± 0.000 for the clearance between the hold and the wall, and 5.000 ± 0.000 for the deformation of the hold. For comparison, a condition with a larger clearance (4.4–5.0 mm) resulted in lower usability scores in a separate evaluation with nine athletes, suggesting that keeping the clearance small is important for maintaining usability.

These results indicate that the designed clearance is sufficiently small to avoid impairing usability, while still preventing direct contact between the hold and the wall under the tested loading conditions. Therefore, the validity of the proposed clearance design for practical force measurement in speed climbing was confirmed.

### 4.2. Force Measurement Performance

#### 4.2.1. Measurement Accuracy Under Static Loads

**Figure 9:**
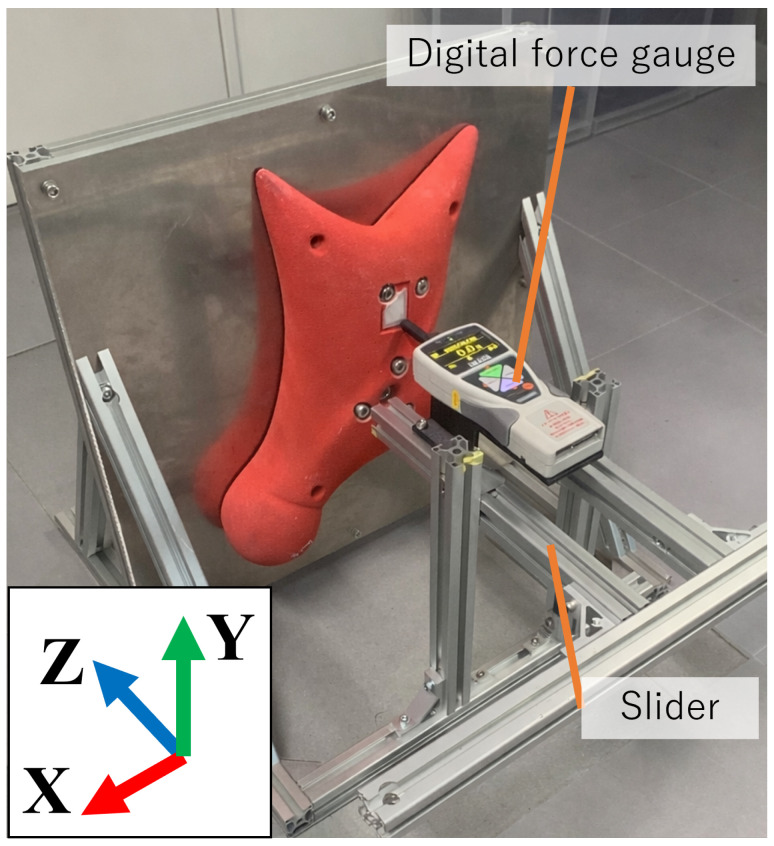
Experimental setup.

**Figure 10:**
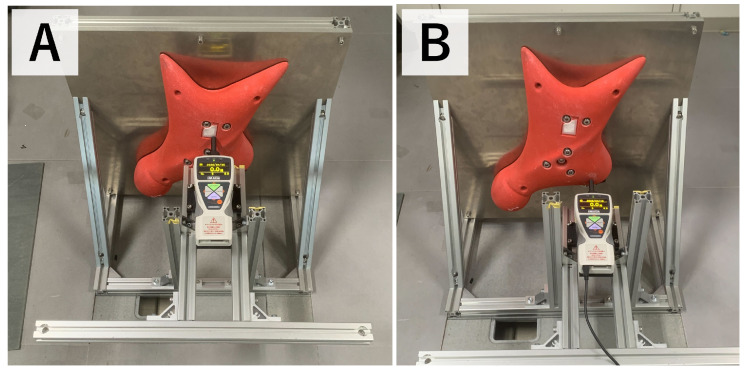
Loading points used in the force measurement accuracy experiment. The left and right loading points correspond to Pattern (**A**) and Pattern (**B**), respectively. External loads were applied to the hold using a digital force gauge.

To evaluate the accuracy of the proposed measurement system, an experiment was conducted in which external loads were applied to the hold using a digital force gauge. Figure 9 shows the experimental setup. A slider mechanism was prepared to allow the digital force gauge to apply force only in the horizontal direction. In this experiment, the hold was pressed at two points, as shown in Figure 10. The loading point on the left side of Figure 10 is referred to as Pattern A, and the loading point on the right side is referred to as Pattern B. The applied force measured by the digital force gauge was compared with the force measured by the embedded six-axis force sensor. The loading conditions in this experiment were selected to provide stable and repeatable reference measurements using the digital force gauge.

In addition to comparing the force magnitude, the experiment was also used to verify whether the estimated point of force application corresponded to the actual pressed point on the hold. Because the six-axis force sensor measures not only force but also moment, accurate estimation of the point of force application requires both the force and moment measurements to be valid. Therefore, agreement between the actual loading point and the estimated point indicates that the proposed system can appropriately measure not only the applied force but also the associated moment and the location of the point of force application.

**Figure 11:**
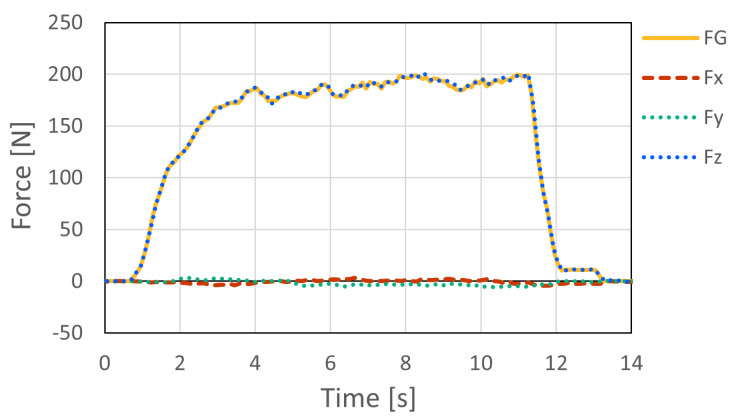
Comparison of the outputs of the six-axis force sensor and the digital force gauge for Pattern A in the force measurement accuracy experiment.

**Figure 12:**
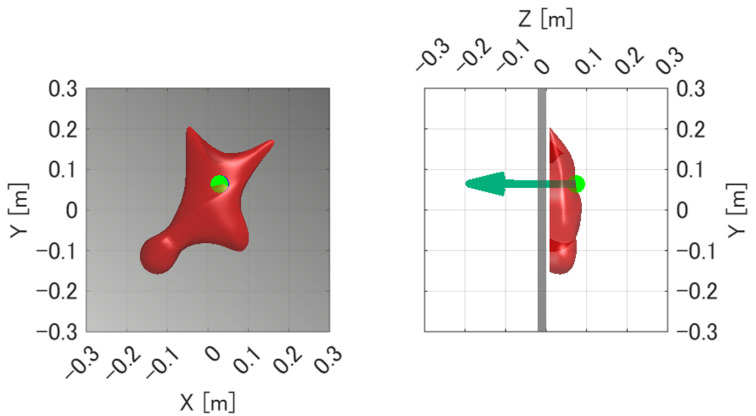
Estimated point of force application for Pattern A visualized on the hold surface. The green dot indicates the estimated point of force application, and the arrow indicates the measured force vector. The estimated point is shown at a location close to the actual loading point.

Figure 11 and Figure 12 show the force measurement result and the visualized point of force application for Pattern A, respectively. The z-axis output, which corresponds to the loading direction of the force gauge, showed good agreement with the force gauge measurement. In addition, the x- and y-axis outputs were nearly zero, confirming that the load was applied appropriately in the horizontal direction. The estimated point of force application was visualized at a location close to the actual loading point.

**Figure 13:**
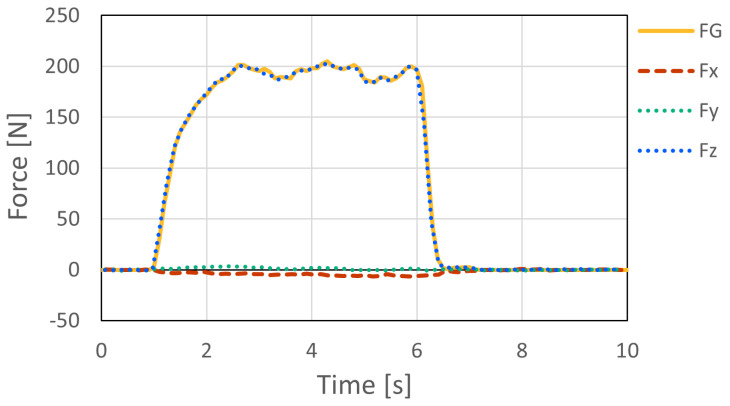
Comparison of the outputs of the six-axis force sensor and the digital force gauge for Pattern B in the force measurement accuracy experiment.

**Figure 14:**
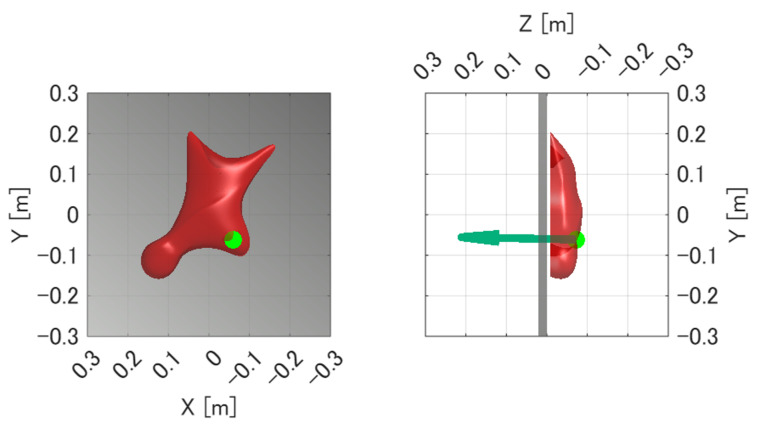
Estimated point of force application for Pattern B visualized on the hold surface. The green dot indicates the estimated point of force application, and the arrow indicates the measured force vector. The estimated point is shown at a location close to the actual loading point.

Similarly, Figure 13 and Figure 14 show the force measurement result and the visualized point of force application for Pattern B, respectively. As in Pattern A, the z-axis output showed good agreement with the force gauge measurement, while the x- and y-axis outputs remained nearly zero. In addition, the estimated point of force application was visualized at a location close to the actual loading point.

For each of the two loading patterns, the experiment was repeated three times. The distance error between the true loading point and the mean estimated point of force application was 1.58 mm for Pattern A and 3.70 mm for Pattern B, and the estimation error was within 5 mm in all trials. These results indicate that the proposed system can measure the applied force and estimate the point of force application with sufficient accuracy under the tested static loading conditions.

#### 4.2.2. Dynamic Response Characteristics Under Impact Loads

While the preceding tests evaluated measurement accuracy under static loading conditions, speed climbing involves highly dynamic and explosive movements. Therefore, a dynamic loading experiment was conducted to evaluate the response characteristics of the proposed system under rapid loading conditions.

**Figure 15:**
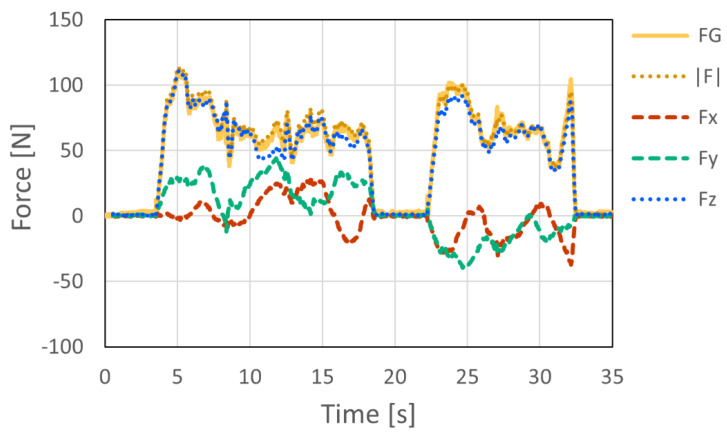
Dynamic force measurement under impact loading. The resultant force $\left|F\right|$ computed from the three-axis outputs of the embedded sensor generally followed the dynamic load measured by the digital force gauge (FG). This test was used mainly to confirm the response tendency under rapid loading.

As shown in Figure 15, the resultant force $\left|F\right|$ computed from the three-axis outputs ($F_{x}$, $F_{y}$, and $F_{z}$) generally followed the dynamic load measured by the digital force gauge (FG). However, the digital force gauge used as the reference device measures force only along a single axis. Therefore, under dynamic loading, slight changes in loading direction, contact condition, and lateral force components may introduce discrepancies between the force measured by the digital force gauge and the resultant force measured by the embedded six-axis sensor. Thus, this experiment was used mainly to confirm the response tendency of the proposed system under rapid loading, rather than to provide a complete dynamic calibration of force magnitude.

The response delay between the digital force gauge and the embedded sensor was within 0–3 ms under the tested conditions. This result indicates that the proposed system has sufficient temporal responsiveness for detecting rapid force changes during speed climbing.

### 4.3. Multi-Sensor Synchronization Accuracy

**Figure 16:**
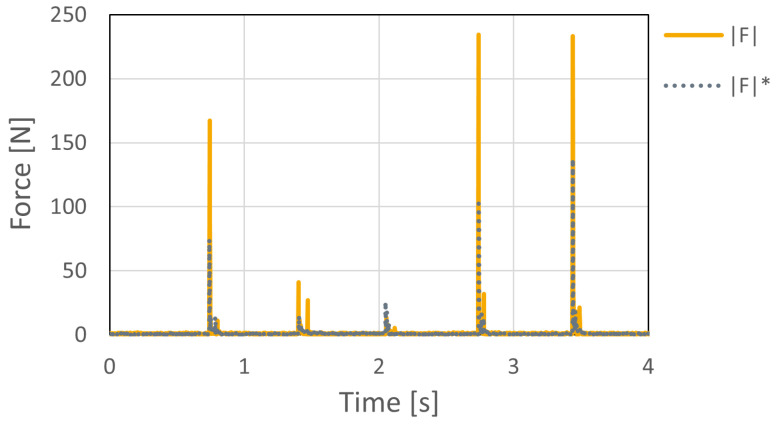
Synchronization accuracy between two instrumented sensors. The resultant forces $\left|F\right|$ and ${\left|F\right|}^{*}$ represent the outputs from the two sensors. The observed synchronization error was within 0–2 ms.

To quantify the synchronization accuracy across multiple instrumented holds, an experiment was conducted using two six-axis force sensors operated as independent measurement units. The sensors were physically stacked and subjected to a simultaneous impulse load. Figure 16 shows the acquired resultant forces, where $\left|F\right|$ and ${\left|F\right|}^{*}$ represent the outputs from the two sensors.

The observed synchronization error between the two devices was within 0–2 ms, which corresponds to only a few sampling periods at the system sampling frequency of 1000 Hz. Considering that the main force variations in speed climbing occur over a substantially longer time scale, this level of synchronization error is sufficiently small for practical force-based analysis.

Therefore, the acquired data from multiple instrumented holds can be directly compared without additional time-delay compensation in the present study.

### 4.4. Measurement Example During Actual Speed Climbing

To verify the feasibility of the proposed system under practical conditions, measurement experiments were conducted on an actual speed climbing wall. The proposed instrumented hold system was installed on Hand 1 and Hand 3 of the official speed climbing route, and the interaction forces exerted by athletes were measured during climbing. Measurements were performed for 16 competitive athletes, including athletes with international- or national-level competition experience (9 males and 7 females), with three climbing trials recorded for each athlete. The purpose of this experiment was to confirm that the proposed system can acquire synchronized force data from multiple instrumented holds under actual speed-climbing conditions.

**Figure 17:**
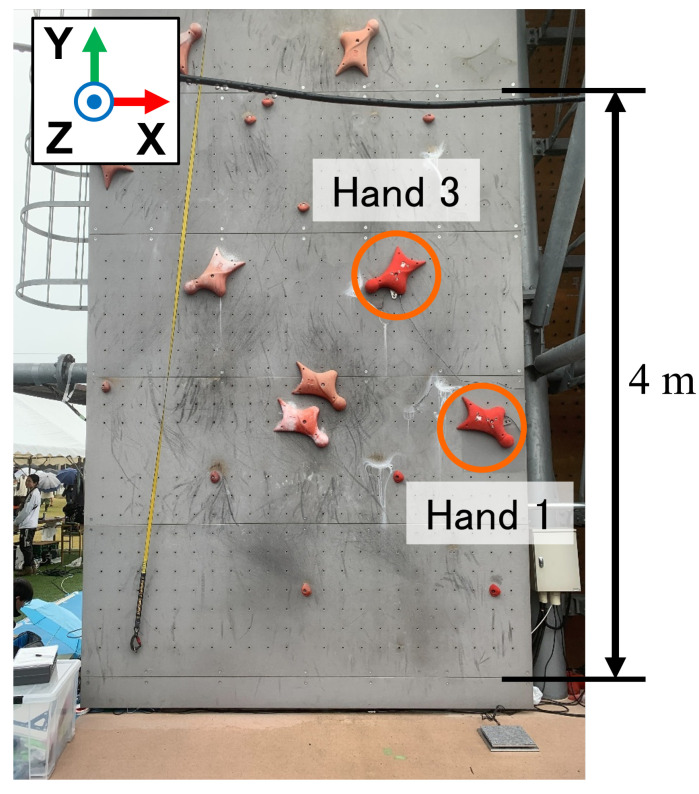
Overview of the measurement experiment on the actual speed climbing wall. The proposed system was installed on Hand 1 and Hand 3. The scale bar indicates 4 m; each wall panel has a height of 1 m.

**Figure 18:**
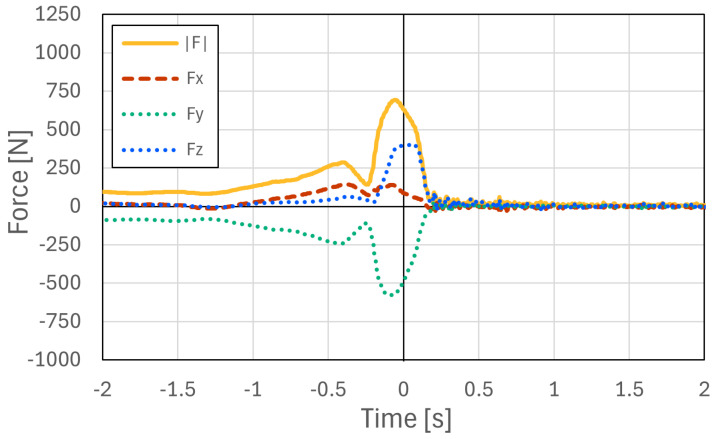
Representative example of the measured three-axis force data at Hand 1 during actual speed climbing, selected from multiple trials. Time zero indicates the instant at which the athlete’s foot left the starting foothold.

**Figure 19:**
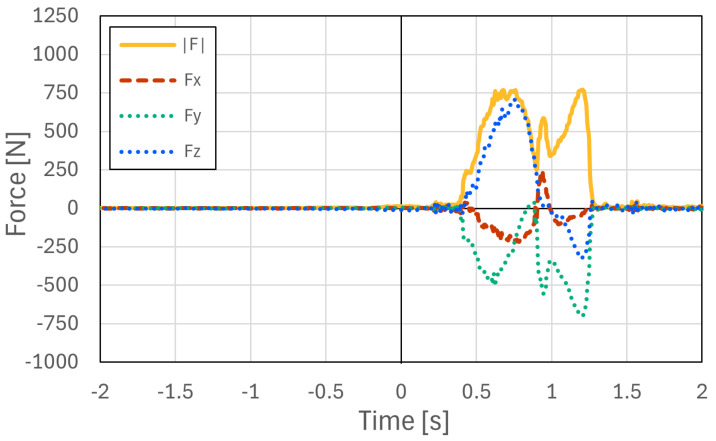
Representative example of the measured three-axis force data at Hand 3 during actual speed climbing, selected from multiple trials. Time zero indicates the instant at which the athlete’s foot left the starting foothold.

An overview of the experimental setup is shown in Figure 17. Figure 18 and Figure 19 show representative examples of the measured three-axis force data at Hand 1 and Hand 3, respectively, selected from the acquired datasets. These two examples were obtained from the same representative climbing trial. In this trial, the athlete started while holding the left side of Hand 1, and the instant at which the athlete’s foot left the starting foothold was defined as 0 s. The athlete then grasped the upper-right region of Hand 3, placed the foot on the left side of Hand 3, and performed a step-up movement. Therefore, the force magnitude at Hand 3 exhibited two peaks, corresponding to the hand contact and subsequent foot loading during the step-up movement. The force data measured at the two holds were synchronized using timestamps recorded during data acquisition. Therefore, the time axes of Figure 18 and Figure 19 share the same temporal reference, allowing direct comparison of the force patterns at the two instrumented holds. In all measured trials, the proposed system successfully acquired force data during actual speed climbing. These results confirm that force measurement can be performed successfully not only in controlled laboratory experiments but also in realistic climbing environments. In actual speed climbing applications, a single run typically lasts only 5–15 s, followed by several minutes of rest. During practical operation, zero-point calibration is performed immediately before each measurement. In addition, the bolts are periodically tightened every few hours as part of a scheduled maintenance procedure, which requires approximately 15 min for detachment and re-installation. This measurement protocol was used to reduce the influence of sensor signal drift and minor structural loosening caused by repeated loading during practical measurement sessions.

### 4.5. Battery Performance

The operating time from full charge to battery depletion was approximately 140–160 min. Charging required approximately 15–20 min. This operating time is sufficient for repeated practical measurements in speed climbing.

## 5. Discussion

The experimental results demonstrated the feasibility of the proposed instrumented climbing hold for force-related measurement in speed climbing. The main contribution of this study is the integration of force and moment measurement, point-of-force-application estimation, and battery-powered cable-free operation into a single instrumented hold designed for standard speed climbing walls. A major practical advantage of the system is that it can be installed on an existing speed climbing wall without modifying the wall structure. This compatibility is achieved by integrating the six-axis force sensor, computation, wireless communication, and power supply inside the hold, thereby providing a self-contained measurement platform for standardized competition walls.

Compared with previously reported climbing force measurement systems, the proposed system provides several practical advantages for deployment on an existing standard speed climbing wall. As summarized in Table 1, the proposed system supports cable-free operation during climbing and does not require structural modification of the wall, external signal or power cables around the hold, or wall-side measurement hardware. In addition, the system directly measures three-axis forces and three-axis moments, enabling point-of-force-application estimation on the hold surface. The experimental results showed that the point-of-force-application estimation error was less than 5 mm under the tested static loading conditions, the response delay under impulse loading was within 0–3 ms, and the synchronization error between two independent sensors was within 0–2 ms. These values provide practical performance indicators for using the system in speed-climbing measurements.

The structural design introduced in this study also contributed to the practical usability of the system. In particular, the clearance between the hold and the wall was introduced to ensure that the external load was transmitted through the sensor, and the fixation structure was reinforced by adopting a two-bolt configuration. These modifications improved the mechanical rigidity of the system while maintaining compatibility with the actual wall environment. The structural evaluation and questionnaire-based usability assessment suggest that the proposed design can preserve measurement validity while maintaining acceptable usability for climbers.

The force measurement experiment under static loading conditions showed that the proposed system can appropriately capture the applied load. The *z*-axis output of the six-axis force sensor agreed well with the force measured by the digital force gauge, while the *x*- and *y*-axis outputs remained nearly zero under horizontal loading. These results indicate that the intended loading condition was properly realized and that the embedded sensor can measure the applied force with sufficient accuracy in the tested range.

The additional dynamic loading tests further demonstrated that the proposed system can capture rapidly changing forces under impact loading. The resultant force measured by the embedded sensor generally followed the dynamic load measured by the digital force gauge, and the response delay was within 0–3 ms under the tested conditions. This delay corresponds to only a few samples at the system sampling frequency of 1000 Hz, indicating that the proposed system has sufficient temporal responsiveness for capturing explosive force changes during speed climbing. In addition, the synchronization experiment using two sensors showed that the the synchronization error between the two devices was within 0–2 ms. These results indicate that the temporal performance of the proposed system is sufficient for capturing and comparing force changes from multiple instrumented holds during actual speed climbing.

Another important finding is that the proposed system was able to estimate the point of force application with reasonable accuracy. The estimated points corresponded closely to the actual loading points, and the estimation error was within 5 mm in all trials. Because the point of force application is estimated from both force and moment data, the estimation accuracy is inherently affected by the metrological characteristics of the six-axis force sensor. The manufacturer-specified characteristics of the sensor, such as non-linearity and cross-axis interference, therefore provide one of the fundamental limits of the estimation accuracy. In addition, the remaining estimation error may also be attributed to uncertainties in the reference loading point, errors in the hold surface model, contact condition variations, and structural deformation of the fixture-hold assembly under load. To reduce high-frequency noise in the force and moment signals, digital low-pass filtering was applied before point-of-force-application estimation. Possible strategies for further reducing these errors include improving the rigidity of the fixture, refining the calibration procedure after assembly, and increasing the accuracy of the three-dimensional hold model. Nevertheless, in the context of speed climbing analysis, the current error of less than 5 mm is sufficiently small for identifying the approximate contact region on the hold, such as finger or foot placement.

The measurements on the actual speed climbing wall demonstrated that the proposed system can acquire synchronized force data from multiple instrumented holds under realistic conditions. In the present study, this experiment was intended primarily to confirm the feasibility of multi-point force measurement during actual climbing, rather than to perform a detailed biomechanical analysis of specific climbing phases. Nevertheless, the successful acquisition of synchronized force data from Hand 1 and Hand 3 indicates that the system can provide a basis for future analyses of the start and early acceleration phases, which have been identified as important for speed climbing performance [1,2].

The practical measurement protocol also supports the applicability of the system in actual speed climbing environments. In speed climbing, a single run typically lasts only 5–15 s, followed by several minutes of rest [2]. In the present system, zero-point calibration is performed immediately before each measurement. In addition, the bolts are periodically tightened every few hours as part of a scheduled maintenance procedure. This protocol helps reduce the influence of sensor signal drift and prevents minor structural loosening caused by repeated loading. A complete long-term cyclic loading test was not conducted in the present study. Therefore, the long-term effects of repeated impact loading on sensor signal drift, bolt loosening, and structural reliability remain to be quantitatively evaluated. Nevertheless, the above procedures help maintain stable operation during practical measurement sessions. Future work should include cyclic loading tests that reproduce repeated climbing impacts, together with pre- and post-test calibration, zero-drift evaluation, and inspection of the fixation structure.

At the same time, several limitations remain. The present system was designed and validated for two standard hand holds used in speed climbing. The basic concept of embedding a six-axis force sensor, electronics, wireless communication, and a battery inside a hold can, in principle, be transferred to other climbing holds if sufficient internal space can be created and if the load path can be designed so that the external force is transmitted through the sensor. However, bouldering and lead climbing use a much wider variety of hold shapes, sizes, orientations, and contact patterns than speed climbing. Therefore, the supporting fixture, internal machining, clearance design, and structural validation would need to be redesigned for each hold type. Consequently, compatibility with bouldering and lead climbing holds should not be assumed without additional mechanical design and validation experiments. In addition, although the present study evaluated static force measurement accuracy, dynamic response under impact loading, and multi-sensor synchronization accuracy, a complete metrological characterization of the proposed device was not performed. In particular, temperature effects and response characteristics under a wider range of loading magnitudes and directions should be evaluated in future work. The validation experiments were conducted under simplified loading conditions using a digital force gauge, and therefore the dynamic and complex interactions that occur during actual climbing were not fully reproduced. The point of force application was evaluated only at two loading points on the hold. The estimation method assumes that the point of force application exists on the surface of the hold, and its performance under more complicated contact conditions, such as distributed contact or multiple simultaneous contact regions, has not yet been examined. A further difficulty is that, under actual climbing conditions, there is no established method for obtaining reliable ground-truth information on the contact state on the hold surface, including the exact contact location, contact distribution, and multiple simultaneous contacts. Therefore, in this study, the proposed method was first validated under simplified loading conditions in which the approximate loading point could be prescribed.

Future work should therefore focus on validation under actual climbing conditions and on further quantitative analysis using the acquired dataset. In particular, it will be important to apply the system during real speed climbing movements and to analyze time-varying force, moment, and point-of-force-application data throughout the start and early acceleration phases [1,2]. Extension of the system to additional hold types, as well as integration with motion capture or video-based analysis, would also broaden its usefulness for biomechanical investigation and training support in speed climbing [7,8].

## 6. Conclusions

In this study, we developed an instrumented climbing hold with an embedded six-axis force sensor for speed climbing. The proposed system integrates sensing, computation, wireless communication, and power supply components inside the hold, enabling force-related measurement without modifying the existing wall structure. This design preserves compatibility with standardized speed climbing walls and improves practical applicability in real climbing environments.

The experimental results confirmed the feasibility of the proposed system. The structural design secured the wall clearance required for measurement and supported practical installation of the system. In the force measurement experiment, the sensor output showed good agreement with the force gauge measurement under controlled loading conditions. Furthermore, the estimated points of force application corresponded closely to the actual loading points, with an error within 5 mm in all trials. These results indicate that the proposed system can measure not only force but also moment and can estimate the point of force application with sufficient accuracy for climbing-related analysis.

The proposed system has the potential to provide detailed information on climber–hold interaction, including the magnitude, direction, and application point of the force. This capability is expected to support more detailed biomechanical analysis of speed climbing than is possible using kinematic information alone. Future work will include validation during actual climbing movements and extension of the system to other hold types used in speed climbing.

## Figures and Tables

**Table 1:** Comparison of the proposed system with related climbing force measurement systems. “Yes” indicates that the feature is supported, and “No” indicates that it is not supported or not reported.

Study	Existing Speed Wall Without Mod.	Cable-Free Operation	Force	Moment	PoFA/ COP
Fuss and Niegl [6]	No	No	Yes	No	COP
Iguma et al. [7]	No	No	Yes	Yes	No
Colombo et al. [8]	No	No	Yes	No	No
Maffiodo et al. [9]	No	No	Yes	No	No
Hayashida et al. [15]	Yes	No	Yes	No	No
Pernus Weber et al. [10]	Yes	No	Yes	No	No
**Proposed system**	**Yes**	**Yes**	**Yes**	**Yes**	**PoFA**

Existing speed wall without mod.: applicable to an existing standard speed climbing wall without structural modification. Cable-free operation: the system can operate during climbing without external signal or power cables around the hold, owing to integrated battery power and wireless data transmission. Moment: direct moment measurement. PoFA: point of force application; COP: center of pressure.

**Table 2:** Manufacturer-specified metrological characteristics of the embedded six-axis force sensor used in this study.

Item	Axis/Condition	Specification
Rated capacity	$F_{x}$, $F_{y}$	±1500 N
Rated capacity	$F_{z}$	±3000 N
Rated capacity	$M_{x}$, $M_{y}$	±150 Nm
Rated capacity	$M_{z}$	±120 Nm
Non-linearity	–	±1.0% R.O.
Cross-axis interference	–	±2.0% R.O.
Resolution	DC operation	±1/4000
Sensor frequency	–	1.2 kHz
A/D settling time	–	Approx. 3.3 ms

**Table 3:** Minimum clearances at C1–C3 under each loading condition. Values in parentheses represent the change relative to the initial clearance.

Loading Point	Load [N]	C1 [mm]	C2 [mm]	C3 [mm]
F1 (push)	50	1.2 (−0.2)	2.2 (−0.1)	2.8 (+0.1)
F2 (push)	50	1.3 (−0.1)	2.1 (−0.2)	2.7 (0.0)
F3 (push)	50	1.4 (0.0)	2.3 (0.0)	2.6 (−0.1)
F4 (pull)	150	1.6 (+0.2)	2.6 (+0.3)	2.5 (−0.2)

## Data Availability

The data presented in this study are not publicly available due to privacy and ethical considerations but are available from the corresponding author upon reasonable request.
